# ChatGPT-4o, Gemini Advanced and DeepSeek R1 in preoperative decision-making for thyroid surgery: a comparative assessment with human surgeons

**DOI:** 10.3389/fonc.2025.1590230

**Published:** 2025-10-24

**Authors:** Long Zou, Peng Zhang, Yu-qi Jiang, Xiao-wen Wang, Xi-jing Yan, Jie-zhong Wu, Jia Qi, Wen-chao Li, Qing-qing Cai, Zhi-rong Xuan, Kun-peng Hu

**Affiliations:** Department of Thyroid and Breast Surgery, The Third Affiliated Hospital of Sun Yat-sen University Lingnan Hospital, Guangzhou, China

**Keywords:** thyroid surgery, large language models, preoperative decision-making, clinical concordance, artificial intelligence

## Abstract

The integration of large language models (LLMs) into surgical decision-making is an emerging field with potential clinical value. This study assessed the preoperative decision-making consistency of ChatGPT-4o, Gemini Advanced, and DeepSeek R1 in comparison with expert consensus, using clinical data from 123 patients undergoing thyroid surgery. Overall concordance rates were 47.97% for ChatGPT-4o, 24.39% for Gemini Advanced, and 56.10% for DeepSeek R1. In thyroidectomy extent decisions, all three models showed moderate consistency with the surgical team, with agreement rates of 61.79% (κ=0.484) for ChatGPT-4o, 67.48% (κ=0.548) for Gemini, and 67.48% (κ=0.535) for DeepSeek R1 (all p < 0.001). However, significant divergence was observed in lymph node dissection planning: ChatGPT-4o achieved a high concordance rate of 69.11% (κ=0.616), DeepSeek R1 showed the highest at 79.67% (κ=0.741), while Gemini’s performance was relatively poor at 34.96% (κ=0.188). Though our findings demonstrate that ChatGPT-4o and DeepSeek R1 exhibit substantial agreement with experienced surgeons in preoperative planning, overall performance still leaves room for improvement. Nevertheless, model-specific variability—particularly in oncologic decision-making—highlights the need for refinement and robust clinical validation before widespread clinical adoption.

## 1 Introduction

Thyroid nodules are a common endocrine disorder with an increasing global incidence (1, 2). While the majority of nodules are benign, approximately 5-15% are malignant, most commonly presenting as differentiated thyroid carcinomas, such as papillary and follicular types (3). The prognosis of thyroid cancer is generally favorable, with a 5-year survival rate approaching 99% (1). However, improper management can result in recurrence or metastasis, significantly impacting patient outcomes. Accurate management is therefore essential to prevent both overtreatment and undertreatment (4).

The management of thyroid nodules involves a comprehensive evaluation, including clinical assessment, imaging studies, and fine-needle aspiration (FNA) cytology, to stratify the malignancy risk and guide therapeutic decisions (5, 6). Treatment options range from active surveillance to various surgical approaches, depending on the malignancy risk. Surgery remains the primary treatment for malignant or suspicious nodules, typically involving thyroidectomy and neck lymph node dissection, with the extent of the procedure determined by factors such as nodule characteristics, extrathyroidal extension, and lymph node involvement (7–10). Despite well-established guidelines, variability in surgical recommendations persists, driven by differences in the interpretation of risk factors and clinician experience. Additionally, thyroid multidisciplinary teams are occasionally incomplete, and increased patient volume alongside limited time often hinders the thorough evaluation of each patient’s clinical data. These factors may affect the consistency of surgical decision-making and potentially lead to suboptimal patient management (11).

Recent advancements in Artificial intelligence (AI), LLMs such as ChatGPT-4o, Gemini Advanced and DeepSeek R1, hold promise in reducing variability among surgeons by providing standardized, evidence-based recommendations, and to serve as efficient tools for medical decision support (12, 13). Yao et al. constructed an AIGC-CAD model and trained it with US images and diagnostic reports, assisting in assessing the risk of thyroid nodules. Lee deployed a LLM to extract key histologic and staging information from surgical pathology reports and received a concordance of 89% with experienced human reviewers (14, 15). While LLMshas demonstrated value in thyroid diagnostic imaging and pathology interpretation (16, 16), its role in aiding complex surgical decision-making for thyroid tumors remains underexplored. Surgical decisions for thyroid cancer require a delicate balance between oncologic control and preservation of thyroid function, making it critical to investigate whether LLMs can replicate or improve human decision-making.

The variability in surgical recommendations and the complexity of thyroid nodule management highlight the need for standardized decision-support tools. This study demonstrates the clinical promise of AI-assisted preoperative decision-making by evaluating and comparing the performance of ChatGPT-4o, Gemini Advanced, and DeepSeek R1 against an expert thyroid surgeon team.

## 2 Materials and methods

### 2.1 Study population

This retrospective study included 123 patients diagnosed with thyroid nodules at the Third Affiliated Hospital of Sun Yat-sen University from February 1, 2024, to April 30, 2024. The inclusion criteria were: (1) age >18 years; (2) confirmed diagnosis of benign or malignant thyroid nodules via FNA biopsy or postoperative histopathology; (3) initial surgical treatment during the study period; (4) complete clinical data, including preoperative evaluations, surgical decisions, and pathology reports; and (5) absence of severe comorbidities. Patients with undifferentiated thyroid carcinoma or significant cardiovascular diseases that posed high surgical risks were excluded.

### 2.2 Data collection

**Figure 1:**
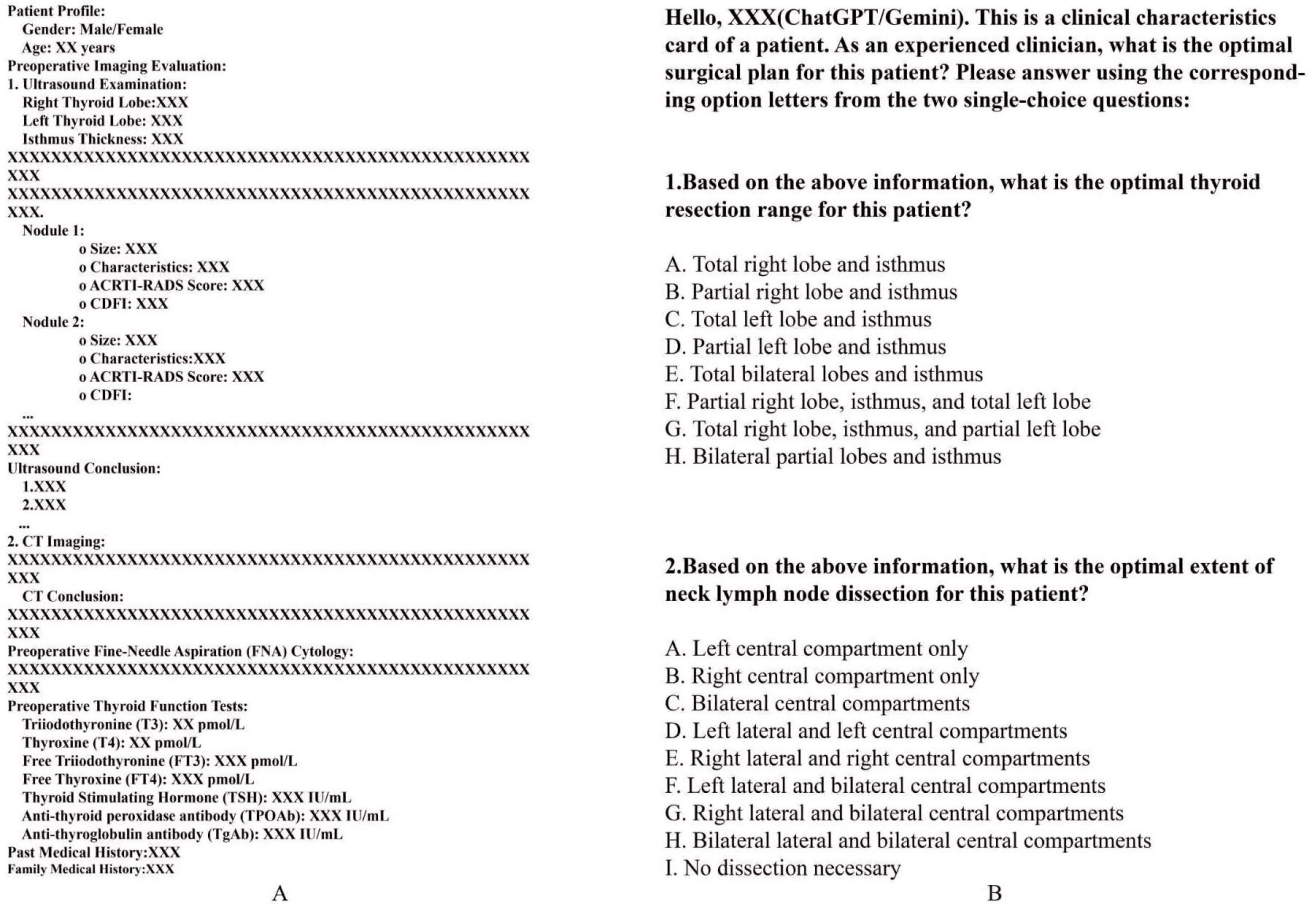
**(A)** Template of sCIC; **(B)** Questions for ChatGPT-4o, Gemini Advanced and DeepSeek R1.

Detailed clinical data were collected for each patient, including demographic information, thyroid function test results, preoperative imaging (neck ultrasound and neck CT), FNA cytology findings, and relevant thyroid-related medical and family history. All preoperative information was consolidated into standardized Clinical Information Cards (sCIC) for subsequent analysis and evaluation of surgical decision-making (**Figure 1A**). The postoperative paraffin pathology results were collected to differentiate between benign and malignant nodules.

### 2.3 sCIC for LLMs analysis and thyroid surgeon team preoperative discussions

Each standardized clinical feature card was independently input into the ChatGPT-4o, Gemini Advanced and DeepSeek R1 models to simulate clinical surgical decision-making. The models were queried with the same question as the clinical team: “What is the optimal surgical strategy for this patient?” To ensure consistency and reliability, the LLMs’ responses were constrained to two single-choice questions (**Figure 1B**). Additionally, the models were required to generate two consecutive identical surgical recommendations. If the responses were consistent, the recommendation was considered the model’s final decision. If the responses differed, the model re-evaluated the case until a consistent decision was achieved. The decision-making processes of each model were conducted independently, without influence from other patient data. Simultaneously, the thyroid surgeon team, consisting of one senior surgeon and four mid-level surgeons, reviewed the clinical feature cards for each patient. **Supplementary Table 1** provides a comprehensive summary of the baseline characteristics of the overall study population. The team conducted preoperative discussions and responded to the same questions posed to the LLMs, in accordance with the second edition of *the Guidelines for the Diagnosis and Treatment of Thyroid Nodules and Differentiated Thyroid Cancer*. Specifically, all five surgeons independently reviewed each case using standardized clinical cards. In cases of disagreement, majority voting was used, and if consensus was not reached, the decision of the senior surgeon served as the final resolution.

### 2.4 Decision comparison and outcome measures

**Figure 2:**
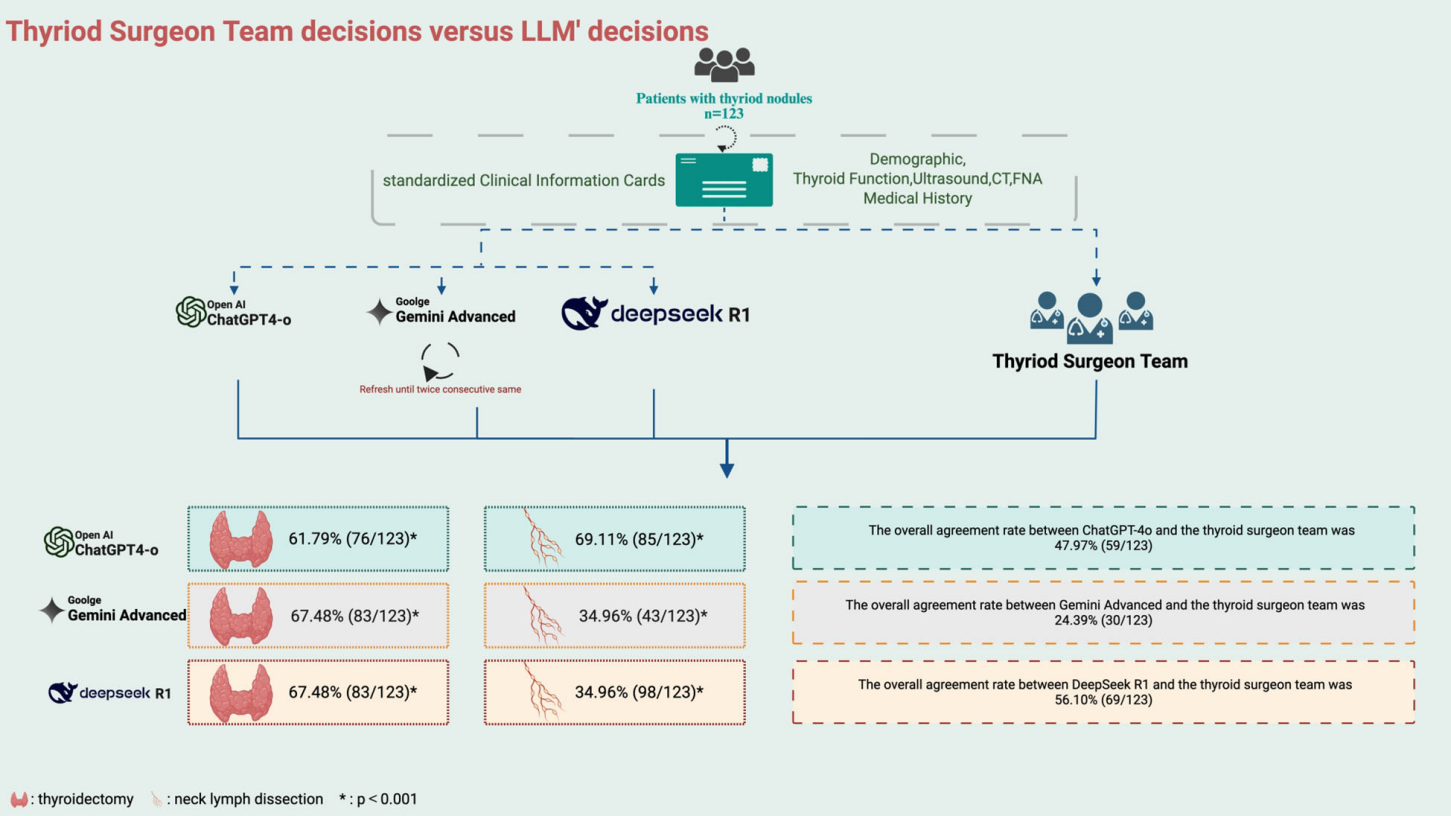
Workflow diagram of agreement rate between thyroid specialist team and LLMs decisions.

The LLMs’ preoperative surgical decisions, defined by two consecutive consistent responses, were compared with the corresponding decisions of the thyroid surgeon team. Surgical decisions were divided into two categories: the extent of thyroidectomy and the extent of lymph node dissection. The primary outcome was the overall agreement rate between the LLMs and the surgeon team regarding thyroidectomy. The secondary outcome was the agreement rate regarding lymph node dissection. Follow-up patient outcomes were not included in this preliminary feasibility study (**Figure 2**).

### 2.5 Statistical analysis

**Figure 3:**
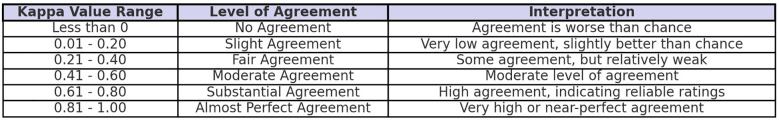
Kappa (κ) value and corresponding level of agreement.

The sample size for McNemar’s test was calculated using G*Power (version 3.1) for paired data with the following parameters: two-tailed test, odds ratio = 0.412, α error probability = 0.05, power (1-β error probability) = 0.8, and proportion of discordant pairs = 0.36, resulting in a required sample size of 123. All statistical analyses were performed using SPSS Statistics (IBM). Descriptive statistics were reported as mean ± standard deviation (SD) for normally distributed continuous variables or median and interquartile range (IQR) for non-normally distributed continuous variables, while categorical variables were summarized as frequencies and percentages. Percentage ratio was used to evaluate concordance in treatment choices among ChatGPT-4o, Gemini Advanced, and the thyroid surgeon team. The Cohen’s Kappa test for unordered categorical data was conducted to evaluate this percentage rate, where κ represents the degree of concordance(**Figure 3**), CI denotes the confidence interval, and p < 0.001 indicates statistical significance. In addition, a *post hoc* power analysis for the observed Cohen’s Kappa values was conducted using a sample size of 123, α = 0.05 (two-tailed), and a null hypothesis κ = 0.2. The results showed that most κ values (≥ 0.484) achieved sufficient power (> 0.8), while κ = 0.188 yielded low power (0.052), consistent with its interpretation of poor agreement.

## 3 Results

### 3.1 Results

#### 3.1.1 Patient characteristics

A total of 123 patients with thyroid nodules who underwent surgical treatment at the Third Affiliated Hospital of Sun Yat-sen University from February 2024 to April 2024 were included in this study. The mean patient age was 45 ± 10 years. According to postoperative paraffin pathology, 83 patients had malignant nodules, while 40 had benign nodules. All patients underwent preoperative thyroid ultrasound and thyroid function tests at our hospital. The mean values for thyroid function were as follows: FT3, 4.3185 ± 0.5004 pmol/L; FT4, 12.9619 ± 1.2140 pmol/L; and TSH, 1.6453 ± 0.8796 µIU/mL. FNA was performed in 80 patients before surgery, yielding consistent results with paraffin pathology: 62 malignant and 18 benign cases. Five patients had a history of hyperthyroidism, one had a history of hypothyroidism, and one had a history of Hashimoto’s thyroiditis; all seven patients had achieved controlled thyroid function preoperatively. Only one patient had a family history of thyroid cancer.

#### 3.1.2 ChatGPT decisions

Preoperative decisions were generated by ChatGPT-4o based on the clinical feature cards of 123 patients. The overall agreement rate between ChatGPT-4o and the thyroid surgeon team was 47.97% (59/123), with a rate of 48.19% (40/83) for malignant nodules and 47.50% (19/40) for benign nodules. In decisions regarding the extent of thyroidectomy, the agreement rate between ChatGPT-4o and the surgeon team was 61.79% (76/123, κ=0.484, CI:[0.368,0.600], p<0.001), with 61.45% (51/83) agreement for malignant nodules and 62.50% (25/40) for benign nodules. For lymph node dissection, the overall agreement rate between ChatGPT-4o and the surgeon team was 69.11% (85/123,κ=0.616, CI:[0.514,0.717], p<0.001), with 67.47% (56/83) agreement for malignant nodules and 72.50% (29/40) for benign nodules.

#### 3.1.3 Gemini decisions

Gemini Advanced provided preoperative decisions for the same cohort of 123 patients. The overall agreement rate between Gemini Advanced and the thyroid surgeon team was 24.39% (30/123), with a rate of 7.23% (6/83) for malignant nodules and 60.00% (24/40) for benign nodules. In decisions regarding the extent of thyroidectomy, the agreement rate between Gemini Advanced and the surgeon team was 67.48% (83/123, κ=0.548, CI:[0.433,0.663], p<0.001), with 65.06% (54/83) agreement for malignant nodules and 72.50% (29/40) for benign nodules. For lymph node dissection, the overall agreement rate between Gemini Advanced and the surgeon team was 34.96% (43/123,κ=0.188, CI:[0.083,0.293], p<0.001), with 13.25% (11/83) agreement for malignant nodules and 80.00% (32/40) for benign nodules. And the extent of lymph node dissection recommended by Gemini was often (79.67%) not greater than that decided by the human surgeons.(**Supplementary Table 1**).

#### 3.1.4 DeepSeek decisions

DeepSeek R1 also generated preoperative recommendations based on the clinical profiles of 123 patients. The overall agreement rate between DeepSeek R1 and the thyroid surgeon team was 56.10% (69/123), with a rate of 59.04% (49/83) for malignant nodules and 50.00% (20/40) for benign nodules. In decisions regarding the extent of thyroidectomy, the agreement rate between DeepSeek R1 and the surgeon team was 67.48% (83/123, κ=0.535, CI:[0.417,0.653], p<0.001), with 65.06% (59/83) agreement for malignant nodules and 72.50% (24/40) for benign nodules. For lymph node dissection, the overall agreement rate between DeepSeek R1 and the surgeon team was 34.96% (98/123, κ=0.741, CI:[0.650,0.832], p<0.001), with 13.25% (65/83) agreement for malignant nodules and 80.00% (33/40) for benign nodules.

### 3.2 Figures, tables and schemes

## 4 Discussion

With the increasing precision required in the management of thyroid nodules, the demand for accurate preoperative decision-making continues to grow. This study provides the first comprehensive evaluation of three advanced large language models (LLMs)—ChatGPT-4o, Gemini Advanced, and DeepSeek—in assisting surgical decision-making for thyroid nodules. Based on data from 123 patients and using only six preoperative clinical features, we demonstrated the feasibility of integrating LLMs into initial surgical assessment. In terms of overall concordance with the thyroid surgeon team, DeepSeek outperformed the others (56.10%), followed by ChatGPT-4o (47.97%) and Gemini Advanced (24.39%). However concordance alone does not equate to clinical benefit. Our study did not assess patients’ outcomes, such as recurrence and complications, so it might serve as a direction for future research.

For decisions regarding the extent of thyroidectomy, all three models showed comparable accuracy, with rates of 61.79% for ChatGPT-4o and 67.48% for both Gemini Advanced and DeepSeek. However, in determining the extent of lymph node dissection, performance varied considerably. DeepSeek achieved the highest concordance (79.67%), followed by ChatGPT-4o (69.11%), whereas Gemini Advanced showed a notably lower concordance (34.96%), indicating a more conservative approach to lymph node management.

Notably, DeepSeek maintained consistent agreement across both malignant and benign cases. For malignant thyroid nodules, ipsilateral central lymph node dissection was consistently recommended, even in cases without evident preoperative metastasis. In benign nodules, lymph node dissection was often deemed unnecessary (17, 18). To further investigate the conservative bias observed in Gemini Advanced, patients were stratified into malignant and benign groups based on postoperative paraffin pathology. ChatGPT-4o demonstrated stable performance in both groups (49.40% for malignant and 47.50% for benign nodules), while DeepSeek showed higher concordance in malignant cases (59.04%) and moderate agreement in benign cases (50.00%). In contrast, Gemini Advanced achieved 60.00% concordance for benign nodules but only 7.23% for malignant ones. In lymph node dissection decisions, Gemini’s concordance was 13.25% for malignant nodules, compared to 80.00% for benign nodules, reinforcing concerns about undertreatment in oncologic scenarios. Regarding the cut-off dates of each LLM’s knowledge, ChatGPT-4o’s was in June of 2024 while DeepSeek R1’s was in July of 2024. However the cut-off date for Gemini Advanced was not stated in any official documentation. Given its release date in February 2024 and industry standard, we propose its training data likely extends through early 2024. Although they have different cut-off dates, all 3 LLMs feature internet connectivity capabilities, enabling them to access the current clinical guidelines. So it might not have much effect on their decision-making.

This study utilized structured standardized clinical information cards (sCICs) with six essential preoperative variables to minimize confounding. Notably, some patients did not undergo fine-needle aspiration (FNA) or CT, not due to missing data but based on clinical discretion. According to international guidelines, omitting these tests in low-risk benign nodules is acceptable. Including such cases allowed for evaluation of the models’ adaptability in real-world conditions where clinical data may be selectively available.

The reliability of AI-generated responses is crucial for clinical applicability. Prior studies offer various methods for response validation, such as using the initial answer as the final decision (Salihu et al.) (19), comparing repeated responses for reproducibility (Yeo et al.) (20), or generating multiple answers to compute an average score (Rao et al.) (21). However, relying on multiple LLMs outputs may potentially lead to a repetitive cycle, with no clear guidance on the ideal number of iterations needed. To address this issue, we continued prompting the LLMs after the initial response was generated, refreshing the output until two identical consecutive answers were obtained. This result was then considered the model’s final decision. We propose that this iterative process functions as a form of introspection, allowing the model to refine its output and enhance reliability. This is based on the premise that large language models are inherently capable of self-verification (22) and serve as increasingly effective reasoners by leveraging internal consistency mechanisms. While this study closely simulates realistic clinical scenarios, incorporating additional thyroid specialists into the discussion panel would undoubtedly enhance the robustness of the findings. Additionally, patient involvement in the final treatment decision is essential, especially in surgeries impacting quality of life and functional outcomes (23, 24). Evidence suggests that patient preferences play a critical role in treatment decisions, and LLMs applications in healthcare should incorporate this aspect to support shared decision-making (SDM). This could enhance LLMs’ utility not only in aiding clinical decisions but also in educating patients about their treatment options, thereby increasing patient engagement and trust in AI-supported medical care.

In fact, as AI integration within clinical workflows is explored further, the outcomes have proven less favorable than the optimistic projections promoted by commercial companies. As highlighted in previous research, the accuracy of responses generated by AI models is a matter of concern. Factors such as the specific version of the language model, limited information regarding user identity, variations in question phrasing, and repeated inquiries can significantly affect the outputs (25). Moreover, these models are prone to generating incorrect or fabricated information, a phenomenon referred to as “artificial hallucination” (26–28). Despite its demonstrated potential, AI decision-making is complex and influenced by various factors, often functioning as an opaque “black box.” This opacity could undermine clinician confidence in AI. Therefore, clinical judgment remains critical, and clinicians should exercise caution and critical thinking when interpreting AI-generated clinical recommendations.

## 5 Conclusions

The demand for advanced, efficient, and precise medical support tools is steadily growing in the management of thyroid nodules. This study highlights the potential of DeepSeek R1, ChatGPT-4o and Gemini Advanced in providing valuable support for preoperative decision-making in thyroid nodules. DeepSeek R1 demonstrated the strongest alignment with surgical team decisions, followed by ChatGPT-4o, then Gemini Advanced. LLMs hold potential as efficient and accessible tools for decision support, but their overall performance still leaves room for improvement.

## Data Availability

The original contributions presented in the study are included in the article/**Supplementary Material**. Further inquiries can be directed to the corresponding author.

## References

[1] Siegel RL Giaquinto AN Jemal A. Cancer statistics, 2024. CA Cancer J Clin. (2024) 74:12–49. doi: 10.3322/caac.21820, PMID: 38230766

[2] Uppal N Collins R James B. Thyroid nodules: Global, economic, and personal burdens. Front Endocrinol. (2023) 14. doi: 10.3389/fendo.2023.1113977, PMID: 36755911 PMC9899850

[3] Alexander EK Cibas ES. Diagnosis of thyroid nodules. Lancet Diabetes Endocrinol. (2022) 10:533–9. doi: 10.1016/S2213-8587(22)00101-2, PMID: 35752200

[4] Kobaly K Kim CS Mandel SJ. Contemporary management of thyroid nodules. Annu Rev Med. (2022) 73:517–28. doi: 10.1146/annurev-med-042220-015032, PMID: 34416120

[5] Wong R Farrell SG Grossmann M. Thyroid nodules: diagnosis and management. Med J Aust. (2018) 209. doi: 10.5694/mja17.01204, PMID: 29996756

[6] Popoveniuc G Jonklaas J. Thyroid nodules. Med Clin North Am. (2012) 96:329–49. doi: 10.1016/j.mcna.2012.02.002, PMID: 22443979 PMC3575959

[7] Burman KD Wartofsky L. CLINICAL PRACTICE. Thyroid nodules. New Engl J Med. (2015) 373. 10.1056/NEJMcp1415786 26650154

[8] Durante C. The diagnosis and management of thyroid nodules: A review. JAMA. (2018) 319. doi: 10.1001/jama.2018.0898, PMID: 29509871

[9] Schneider DF Chen H. New developments in the diagnosis and treatment of thyroid cancer. CA Cancer J Clin. (2013) 63:374–94. doi: 10.3322/caac.21195, PMID: 23797834 PMC3800231

[10] Sherman SI. Thyroid carcinoma. Lancet. (2003) 361:501–11. doi: 10.1016/S0140-6736(03)12488-9, PMID: 12583960

[11] Doubleday A Sippel RS. Surgical options for thyroid cancer and post-surgical management. Expert Rev Endocrinol Metab. (2018) 13. doi: 10.1080/17446651.2018.1464910, PMID: 30058897

[12] Lee P Bubeck S Petro J. Benefits, limits, and risks of GPT-4 as an AI chatbot for medicine. N Engl J Med. (2023) 388:1233–9. doi: 10.1056/NEJMsr2214184, PMID: 36988602

[13] Haug CJ Drazen JM. Artificial intelligence and machine learning in clinical medicine, 2023. N Engl J Med. (2023) 388:1201–8. doi: 10.1056/NEJMra2302038, PMID: 36988595

[14] Yao J. Multimodal GPT model for assisting thyroid nodule diagnosis and management. NPJ Digital Med. (2025) 8:245. doi: 10.1038/s41746-025-01652-9, PMID: 40319170 PMC12049458

[15] Lee D. Using large language models to automate data extraction from surgical pathology reports: retrospective cohort study. JMIR Form Res. (2025) 9:e64544–4. doi: 10.2196/64544, PMID: 40194317 PMC11996145

[16] Wu S-H. Collaborative enhancement of consistency and accuracy in US diagnosis of thyroid nodules using large language models. Radiology. (2024) 310:e232255. doi: 10.1148/radiol.232255, PMID: 38470237

[17] Haugen BR. 2015 american thyroid association management guidelines for adult patients with thyroid nodules and differentiated thyroid cancer: the american thyroid association guidelines task force on thyroid nodules and differentiated thyroid cancer. Thyroid. (2016) 26:1–133. doi: 10.1089/thy.2015.0020, PMID: 26462967 PMC4739132

[18] Lebbink CA. 2022 European Thyroid Association Guidelines for the management of pediatric thyroid nodules and differentiated thyroid carcinoma. Eur Thyroid J. (2022) 11:e220146. doi: 10.1530/ETJ-22-0146, PMID: 36228315 PMC9716393

[19] Salihu A. A study of ChatGPT in facilitating Heart Team decisions on severe aortic stenosis. EuroIntervention. (2024) 20:e496–503. doi: 10.4244/EIJ-D-23-00643, PMID: 38629422 PMC11017225

[20] Yeo YH. Assessing the performance of ChatGPT in answer- ing questions regarding cirrhosis and hepatocellu- lar carcinoma. Clin Mol Hepatol. (2023). doi: 10.3350/cmh.2023.0470, PMID: 36946005 PMC10366809

[21] Rao A. Assessing the utility of chatGPT throughout the entire clinical workflow: development and usability study. J Med Internet Res. (2023) 25:e48659. doi: 10.2196/48659, PMID: 37606976 PMC10481210

[22] Weng Y. Large language models are better reasoners with self-verification. (2023). doi: 10.48550/arXiv.2212.09561

[23] Yang W Lee YK Lorgelly P Rogers SN Kim D. Challenges of shared decision-making by clinicians and patients with low-risk differentiated thyroid cancer: A systematic review and meta-ethnography. JAMA Otolaryngol Head Neck Surg. (2023) 149:452–9. doi: 10.1001/jamaoto.2023.0101, PMID: 36951823

[24] Kaul P Kumar Garg P. Enabling patient empowerment in treatment decisions for thyroid cancer. JAMA Surg. (2024) 159. doi: 10.1001/jamasurg.2023.3951, PMID: 37672280

[25] Salihu A. Towards AI-assisted cardiology: a reflection on the performance and limitations of using large language models in clinical decision-making. EuroIntervention. (2023) 19:e798–801. doi: 10.4244/EIJ-D-23-00461, PMID: 38050992 PMC10687640

[26] Alkaissi H McFarlane SI. Artificial hallucinations in chatGPT: implications in scientific writing. Cureus. (2023) 15:e35179. doi: 10.7759/cureus.35179, PMID: 36811129 PMC9939079

[27] Ji Z. Survey of hallucination in natural language generation. (2024). doi: 10.48550/arXiv.2202.03629

[28] Perez F Ribeiro I. Ignore previous prompt: attack techniques for language models. (2022). doi: 10.48550/arXiv.2211.09527

